# Cognitive Aid for Anesthetic Preparation in An Emergency Situation: A Simulation-Based Study

**DOI:** 10.3390/healthcare9121646

**Published:** 2021-11-27

**Authors:** Emmanuel Rineau, Anna Collard, Lorine Jean, Sarah Guérin, Louise Maunoury, Ludovic Martin, Sigismond Lasocki, Maxime Léger

**Affiliations:** 1 All’Sims Center for Medical Simulation, Health Faculty and University Hospital of Angers, 49100 Angers, France; anna.collard@live.fr (A.C.); Lorine.Jean@chu-angers.fr (L.J.); sarah.guerin@chu-angers.fr (S.G.); louise.maunoury@chu-angers.fr (L.M.); LuMartin@chu-angers.fr (L.M.); silasocki@chu-angers.fr (S.L.); Maxime.Leger@chu-angers.fr (M.L.); 2 Department of Anesthesia and Critical Care, University Hospital of Angers, 49100 Angers, France; 3 Department of Dermatology, University Hospital of Angers, 49100 Angers, France

**Keywords:** patient safety, anesthesia, checklist, cognitive aid, emergency, simulation

## Abstract

When anesthesia checklists and preparations are performed urgently, omissions may occur and be deleterious to the patient. The aim of this study was to evaluate in simulation the interest of a cognitive aid to effectively prepare an anesthetic room for an emergency. In a prospective single-center simulation-based study, 32 anesthesia residents had to prepare an anesthetic room in an emergency scenario, without cognitive aid in the first phase. Three months later (phase 2), they were randomly assigned to receive a cognitive aid (aid group) or no additional aid (control) and were involved in the same scenario. The primary outcome was the validation rate of each essential item in the first 5 min in phase 2. Eight items were significantly more frequently completed in the first 5 min in the aid group in phase 2 (vs. phase 1), compared with two only in the control group. However, there were no significant differences in the overall number of completed items between the two groups, as both groups completed significantly more items in phase 2, either in the first 5 min (19 (14–23) vs. 13 (9–15) in phase 1 for all residents, *p* < 0.001) or without time limit. Preparation times were reduced in phase 2 in both groups. In conclusion, the use of a cognitive aid allowed anesthesia residents to complete some safety items of a simulated urgent anesthesia preparation more frequently. In addition, despite daily clinical experience, a single simulation session improved anesthesia preparation and reduced the preparation time with or without cognitive aid.

## 1. Introduction

Checklists such as the “World Health Organization Surgical Safety Checklist” are simple and efficient reproducible tools designed to optimize the safety of some high-risk procedures [1,2]. The “anesthetic checklist” is a list of safety items to verify before a procedure under sedation, general or regional anesthesia. Most of the time, it is combined with the preparation of material and medications required for the anesthesia; and Machine (ventilator), Suction, Monitor, Airways, Intravenous lines, Drugs and Safety, which form the widely used mnemonic MSMAID, are the common parts of the checklist [3,4,5,6]. Although this checklist is done almost daily, before each elective or urgent procedure, there is no precise and unified description in some centers, especially in France, unlike in some countries such as United Kingdom and Spain [3,4]. The checklist is generally taught at the start of anesthesia residency with theoretical lessons followed by everyday practice during internships.

The anesthetic preparation has to sometimes to be performed urgently and faster, in a non-prepared room or in an unfamiliar or unusual environment (e.g., locum in an unknown hospital or anesthesia in a catheterization laboratory). In these emergency situations, stress and lack of time can interfere with reflection and judgement, and quickly lead to omissions, errors, and serious adverse events [7]. The use of checklists in the form of cognitive aids is therefore particularly useful, especially in critical anesthetic situations [8,9]. These aids are built to guide the physician by listing validated steps to manage a specific situation, in order to avoid cognitive errors and improve patient management [10].

For several years, simulation has been integrated into the training of anesthesia residents [11], as it offers the possibility to acquire skills in conditions close to reality, without risk for the patient [12,13]. Interestingly, the impact of cognitive aids has also been assessed in simulation before their implementation in a real setting [10,14]. However, to our knowledge, the use of simulation and cognitive aids for the anesthesia room preparation has not been studied. The main objective here was to assess whether the use of a cognitive aid was useful to complete checks for more safety items of the anesthetic preparation in a simulated emergency situation.

## 2. Materials and Methods

This prospective simulation-based single-center study was performed between January and June 2020 in the simulation center of Angers University Hospital. The study was approved by the Institutional Review Board (Comité d’Ethique du CHU d’Angers, reference number 2021/095). Participants gave written consent to use their data.

### 2.1. Participants

All anesthesia residents who had previously completed one semester or more in an anesthesia unit, from the first to the fifth year of anesthesia residency, were eligible. All participants had already practised simulation in this center, for other purposes than the anesthesia checklist. Simulation sessions were integrated as part of their continuing education and had no evaluation purpose.

### 2.2. Environment and Conduct of Sessions

The simulation sessions took place inside the simulation center. The room reproduced an operating room (OR) identically in its organization and equipment. The anesthesia ventilator was a Datex Ohmeda Avance^®^ S5 (GE Healthcare, France), as in the Anesthesia Department. Each resident was assessed separately and participated in both phases.

#### 2.2.1. First Phase

##### Briefing

Subjects played their own role as anesthesia residents. They were instructed to prepare the “anesthesia room” in an OR for an emergent surgery (subdural hematoma evacuation at night), before the arrival of the patient and the on-call team (nurse anesthetist and senior anesthetist). The only supplementary information they had was the male gender and the 5-min delay before the patient’s arrival. Instruction was provided to the residents to inform the two observers (present in the room) once their preparation was completed.

##### Simulation and Assessment

Once the timer started, participants had to do their anesthetic verifications and preparations. They were allowed to request additional information concerning the patient or the equipment not directly visible in the room, and observers then provided details orally, according to pre-established standardized responses.

Residents were notified as soon as the first 5 min had elapsed. Observers gave extra indefinite time to the residents with the information that the fictitious patient was still on the way to the OR. The timer was stopped when residents informed the observers that they had finished.

The two instructors, who were observers in the same room, had to report in real time each item carried out by the resident on an evaluation grid, in two separate columns: before or after the first 5 min. The simulation session was not filmed.

##### Debriefing

At the end of the session, a personalized debriefing was conducted by the two observers, in the presence of the participant only. The observers summarized the checklist points correctly completed and suggested improvement clues for the non-validated items and the reasons of their importance.

##### Anxiety

Immediately after the debriefing, participants completed a validated French version of the Spielberger anxiety questionnaire (STAI: State-Trait Anxiety Inventory form) [15,16]. The STAI-YA part was used to assess the level of anxiety related to the simulation scenario. The STAI-YB part was used to assess anxiety as a personality trait (i.e., basal level of anxiety). Each scale rates from 20 to 80/80, with a usual breakdown into five groups according to the level of anxiety, ranking from <36 = very low, to >65 = very high. A score >36 on the Anxiety State Scale was considered significant [17].

#### 2.2.2. Second Phase

Three months later, participants were randomly assigned to one of two groups: one group that received a cognitive aid for the next simulation session (aid group), and one group that did not (control group). They were matched according to the number of validated semesters in anesthesia. Residents of the aid group were informed of the cognitive aid availability during the briefing of the second phase only. The scenario and the timing conditions were identical to those of the first phase. The observers for this second phase were the same observers as in the first phase (A.C. and L.J.) The cognitive aid was given and explained to all participants at the end of each simulation session, including to the residents of the control group.

### 2.3. Exhaustive Checklist and Essential Items

**Figure 1:**
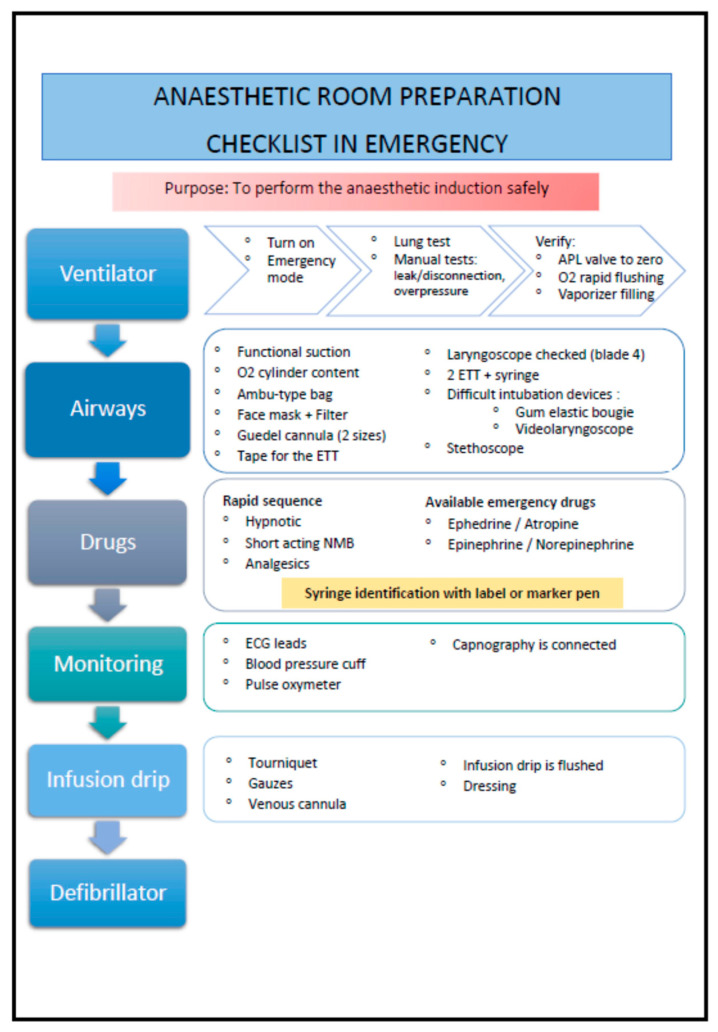
English translation of the cognitive aid given to the anesthesia residents. ECG, electrocardiogram; ETT, endotracheal tube; APL, adjustable pressure-limiting; NMB, neuromuscular blocker.

As no official checklist dedicated to the anesthetic preparation was available in France at the time of our study, we drew up an exhaustive list of 60 items corresponding to essential or non-essential steps for a usual anesthesia room preparation, according to various reports and recommendations from the French Society of Anesthesia and Intensive Care Medicine (SFAR) [18,19], the Royal College of Anesthetists [3,20], and the World Health Organization (WHO) [1]. We then selected 40 items deemed essential for a secure room preparation in an emergency context. These items make up the cognitive aid presented in Figure 1.

### 2.4. Outcomes

The primary objective was to assess the potential efficacy of the cognitive aid to avoid missing essential items of the anesthesia checklist in an emergent situation. The primary outcome was the validation rate of each essential item in the first 5 min in phase 2 in each group.

Secondary objectives were to make an inventory of practices of the anesthetic preparation by anesthesia residents of our University Hospital, and to assess if there was a benefit of the simulation training on the anesthesia preparation. The secondary evaluation criteria were the overall number of completed items in both groups and in both phases, the preparation duration, and the anxiety of the residents.

### 2.5. Statistical Analysis

Data, presented as numbers (percentages) or medians (Q1–Q3), were compared between the two groups and between the two phases using a Fisher test or a Wilcoxon test respectively (JMP software, SAS Institute, France). Numbers of validated essential items and the times to complete the checklist were compared for the same group between the two phases in a paired manner. A *p* value < 0.05 was considered significant.

## 3. Results

### 3.1. Population Characteristics

Of the 51 anesthesia residents of our University Hospital in 2020, 32 residents were included from January to June 2020, 16 in the aid group and 16 in the control group. Nineteen anesthesia residents of our University were not included as they were doing internships in other cities at the time of the study. The Table 1 shows demographic data of included residents.

### 3.2. Primary Endpoint

**Figure 2:**
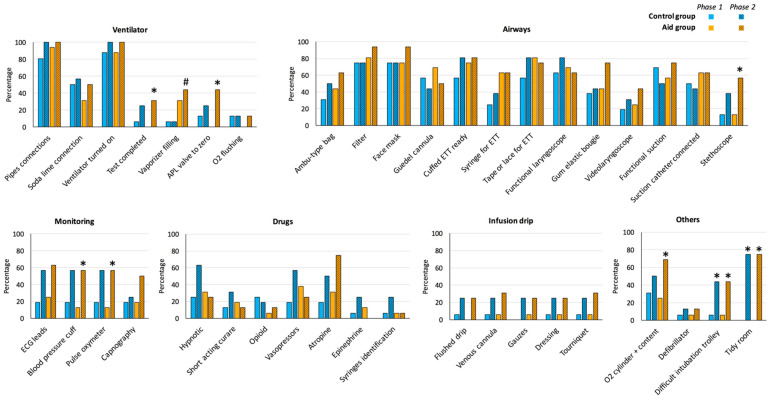
Percentages of residents who validated each required item in the first 5 min in control (blue bars) and aid (yellow bars) groups in phase 1 (plain filling) and phase 2 (hatched filling). *, *p* < 0.05 using a paired comparison with phase 1. *p* < 0.05 between the control group and the aid group in phase 2.

Eight items among the 40 essential items were significantly more frequently completed in the first 5 min in the aid group in phase 2 (in comparison with phase 1), compared with only two in the control group (Figure 2). These eight items were the completion of the ventilator test (mostly by using the emergency mode), the setting of the adjustable pressure-limiting (APL) valve to zero (that was basically set to 50 cm H_2_O), the presences of a stethoscope, a blood pressure cuff, a pulse oximeter, an oxygen cylinder, a difficult airway trolley and tidying up the room as they were preparing it. In the control group, a difficult airway trolley and tidying up the room were the only items more frequently validated in phase 2, in comparison with phase 1.

One item, the vaporizer filling verification, was significantly more frequently completed in phase 2 in the aid group, compared to the control group (Figure 2). For other items, the differences observed between the two groups were not statistically significant.

### 3.3. Number and Details of Completed Items in Both Phases

As shown in Table 2, both groups completed significantly more essential items in phase 2, either within the first 5 min (13 (9–15) vs. 19 (14–23) for overall residents in phase 1 and 2, respectively, *p* < 0.001) or without time limit (30 (25–33) vs. 33 (29–36) for overall residents in phase 1 and 2, respectively, *p* = 0.001), with no significant differences between the 2 groups.

#### 3.3.1. First Phase

Ten items only (out of a possible total of 40 items) were validated by more than half of the residents in phase 1 in the first 5 min, although 33 were validated without time limit in this phase (Figures S1 and S2 of the Supplementary Material). In the vast majority of cases, the ventilator was tested from the timer start (55% within 5 min). However, the duration of the automated test (vs. the emergency mode) only allowed its use beyond the 5 min, resulting in consideration of completing the ventilator test in only 3% of the residents in the first 5 min (vs. 88% without time limit). There were no significant differences between the two groups in phase 1 (Figure 2).

#### 3.3.2. Second Phase

In phase 2, residents of both groups validated 14 items significantly more frequently than in phase 1 in the first 5 min, and five without time limit (Figures S1 and S2 of the Supplemental Material). Interestingly, only nine (56%) residents of the aid group really used the cognitive aid provided by the instructor.

### 3.4. Duration

**Figure 3:**
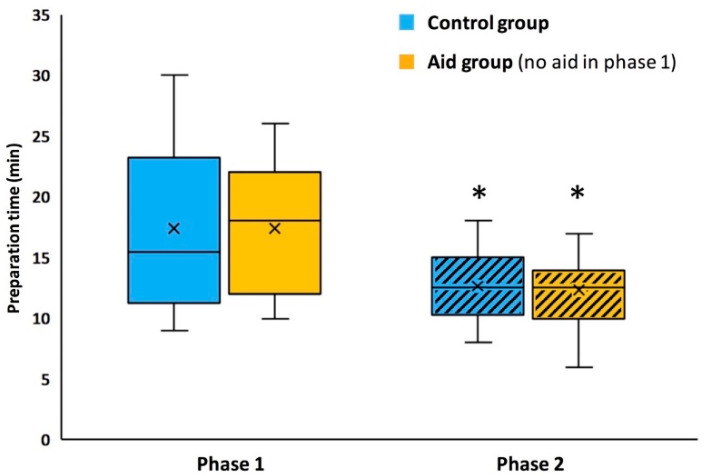
Durations of the anesthetic preparation by residents of control group (blue boxes) and aid group (yellow boxes), in phase 1 (plain filling) and phase 2 (hatched filling). Residents in the aid group had a cognitive aid in phase 2, but not in phase 1. *n* = 16 in each group. *, significant difference with the same group in phase 1.

Durations of the anesthetic room preparation were not significantly different between the two groups in the two phases (Figure 3). However, durations were significantly reduced in both groups between the 2 phases. Thus, 15 (94%) of residents in the aid group and 14 (88%) in the control group finished their OR preparation in less than 15 min in phase 2, vs. 7 (44%) and 8 (50%) in phase 1 (*p* = 0.006 and *p* = 0.05) for aid and control groups, respectively.

### 3.5. Anxiety

The STAI-YB values observed indicated a low level of anxiety at baseline. The STAI-YA score was 28 out of 80 in both groups at the end of the first session, corresponding to a “very low” level of anxiety related to this session. The score was not significantly different in the second phase in both groups (Table 3).

## 4. Discussion

In this prospective simulation-based study, eight safety items were completed significantly more frequently with the use of a cognitive aid for an urgent anesthesia preparation, compared with only two with no aid. However, despite their previous clinical experience, residents of both groups (aid and no aid) improved their performances in terms of number of validated items and time after a single simulation session devoted to the anesthesia checklist.

In phase 1, we observed that many important safety items of the five studied categories (ventilator, airways, drugs, drip, and emergency material) were not validated by more than 50% of the residents, mostly in the first 5 min but also in the unlimited time for some of them. Interestingly, compared with phase 1, the number of completed essential items in phase 2 was significantly greater in both groups, indicating the effect of a single simulation session on this topic. However, even if both groups increased their results, the observed improvement was significant for more items in the aid group. This result was expected and desired as the objective of our cognitive aid was to avoid forgetting some of these items, essential to patient safety (such as the presence of a stethoscope or an oxygen cylinder in the room, or the ability to effectively prepare the ventilator in an emergency situation). The overall number of essential items was however not different between the two groups, as we could have expected it. This result is probably due to the low number of included residents in each group and to the fact that both groups improved their results in phase 2.

Interestingly, the cognitive aid was used by only half of the residents who were in possession of it, which is consistent with the results of a previous study in an emergency anesthesia setting [21]. However, although some residents seemed to forget its availability, a few of them used it for a final check only, which allowed them to correct some omissions. Possible explanations are its format (color-printed on A5 sheet), or its location (on the machine). The ideal design for cognitive support remains unknown, although the paper format seems to be favored [21].

In France, the real use of a cognitive support is not very common in the field of anesthesia, unlike in Anglo-Saxon countries [22]. Indeed, as shown in a recent survey among French and Canadian professionals, only 56% of French anesthetists, compared to 92% of Canadians, were aware of the concept of cognitive aids, and only 40% of them had already used them in a real life [22]. The implementation of a cognitive support in a hospital environment is a complex process and must be part of a multidisciplinary dynamic to be adopted by the greatest number. We could have included anesthetic nurses in our study since they are important participants in the preparation of the OR in France. Otherwise, the development of digital cognitive aids is booming, and numerous learning societies regularly publish online cognitive aids [23], sometimes usable on a smartphone application [24].

In addition, our study suggests that the practice via simulation improves quality and speed of the residents to carry out an emergency anesthetic room preparation. Indeed, while included anesthesia residents were used to perform pre-anesthetic checklists, almost daily, a single simulation session significantly increased the number of validated items, regardless of the presence of a cognitive support. In addition, the duration was significantly reduced by six min in both groups between the two phases, which can be clinically significant in some urgent situations. This result was most likely facilitated by the short-personalized debriefing performed at the end of the first session, in which each resident was notified of the items he had forgotten, and in which the importance of each of these items was explained. This aspect, essential in simulation, is probably less present in everyday life. Although numerous studies have shown the benefits of health simulation in terms of development of medical [25,26] or technical skills [27], especially in the management of rare or critical situation in anesthesia [28,29], its benefit had not been previously assessed, to our knowledge, for the anesthesia checklist. The duration of the positive effect linked to this simulation session, which may be longer than for rarer emergency situations, remains to be evaluated.

Our study has several limitations, including the possible bias linked to the use of the simulation. The OR was reproduced with some but slight differences in organization and location of the anesthetic material that could have affected the habits of residents, and therefore, their performances. However, all residents had already completed at least one session in this simulation center, and they had an overview of the room before the session started. As this scenario remains plausible in real life, for anesthetic procedures in an unknown unit, in a new operating theatre, or in some emergency locations such as the catheter laboratory or the radiology unit, this aid can provide more value. Furthermore, we were expecting a greater difference in the number of completed items between the two groups. Several reasons might explain this lack of significant difference. First, as explained previously, both groups improved their performances in phase 2, probably due to the first simulation session. Second, the number of residents was low and likely impacted the power of the study on this endpoint. Third, the use of the aid was not compulsory, although it was immediately available (as it may be in a real situation) and this aid was not read by residents before the simulation session. Finally, the time chosen for our main criterion was perhaps too short (5 min), preventing the aid group to complete more items. This time limit of 5 min was chosen arbitrarily to exaggerate the urgency of the situation but such a real-life situation remains plausible. Nevertheless, given the proportion of essential items validated in less than 5 min, this period is probably largely insufficient as many essential security items were not checked by more than half of the residents in the first 5 min, thus the reason why an indefinite additional time was systematically given to the resident for completing the preparation, and this additional time allowed us to assess which items were considered as ‘priority’ by the residents.

Recommendations from experts from the main French societies in anesthesia, emergency, and intensive care (SFAR, SRLF*, SFMU*, SoFRaSimS*), recently published in 2020, encourage the integration of procedural and high-fidelity simulation into the initial and continuing medical education, to acquire and maintain technical, clinical, and communication skills [11]. The results of our study should encourage anesthesiologists to integrate such “checklist training sessions” into their educational program.

## 5. Conclusions

In a simulated emergency situation with time restriction, the use of a cognitive aids for the anesthetic preparation of the OR allowed anesthesia residents to verify some important safety items more frequently. Interestingly, a single simulation session was able to significantly improve their speed and the number of validated items, with or without additional cognitive aid. Thus, despite their clinical experience and the almost daily nature of this situation, the anesthesia checklist should probably be part of the simulation training of anesthesia residents.

## Figures and Tables

**Table 1:** Demographic data of the anesthesia residents included in the study.

Demographic Data	Control Group (*n* = 16)	Aid Group (*n* = 16)	*p*
Age, year	27 (25–28)	27 (26–29)	0.54
Sex, male	8 (50%)	11 (69%)	0.47
Anesthesia training level, year	3.5 (2–4)	3 (2–4)	0.65

Data are expressed as numbers (percentages) or medians (Q1–Q3).

**Table 2:** Number of essential items (out of a possible total of 40 items) validated by residents in the first 5 min and without time limit in both phases.

	Phase 1		Phase 2		Paired Comparison Phase 1 vs. 2	
	Control (*n* = 16)	Aid (*n* = 16)	Control (*n* = 16)	Aid (*n* = 16)	*p*, Control Group	*p*, Aid Group
First 5 min	12 (8–15)	13 (10–16)	16 (13–23)	21 (15–22)	0.0011	0.0003
Without time limit	30 (24–33)	30 (27–33)	32 (29–34)	34 (29–38)	0.0381	0.0196

Data are expressed as medians (Q1–Q3). Residents in the aid group had a cognitive aid in phase 2 only (not in phase 1). There were no significant differences between the two groups in each phase.

**Table 3:** Anxiety evaluation in residents using the State-Trait Anxiety Inventory form.

	Phase 1		Phase 2		Paired Comparison Phase 1 vs. 2	
	Control (*n* = 16)	Aid (*n* = 16)	Control (*n* = 16)	Aid (*n* = 16)	*p* for Control Group	*p* for Aid Group
STAI YA	28 (24–32)	28 (25–38)	30 (23–34)	29 (23–32)	0.84	0.37
STAI YB	33 (31–41)	36 (30–39)	35 (31–43)	33 (31–39)	0.67	0.66

Data are expressed as medians (Q1–Q3). Residents in the aid group had a cognitive aid in phase 2 only (not in phase 1). STAI, State-Trait Anxiety Inventory. There were no significant differences between the two groups in each phase.

## Data Availability

The data presented in this study are available on request from the corresponding author.

## Supplementary Materials

The following are available online at https://www.mdpi.com/article/10.3390/healthcare9121646/s1, Figure S1: Percentages of residents (*n* = 32) who validated each required item in the first 5 min, in phase 1 and phase 2; Figure S2: Percentages of residents (*n* = 32) who validated each required item, without time limit, in phase 1 and phase 2.
