# Supplementary material for: Cognitive Aid for Anesthetic Preparation in An Emergency Situation: A Simulation-Based Study
Source: Healthcare (Basel). 2021 Nov 27;9(12):1646. doi: 10.3390/healthcare9121646 (PMC8700863; doi:10.3390/healthcare9121646)

Supplementary Materials

Figure. S1. Percentages of residents (n = 32) who validated each required item in the first 5 minutes, in phase 1 and phase 2. *, p <0.05 between the 2 phases.

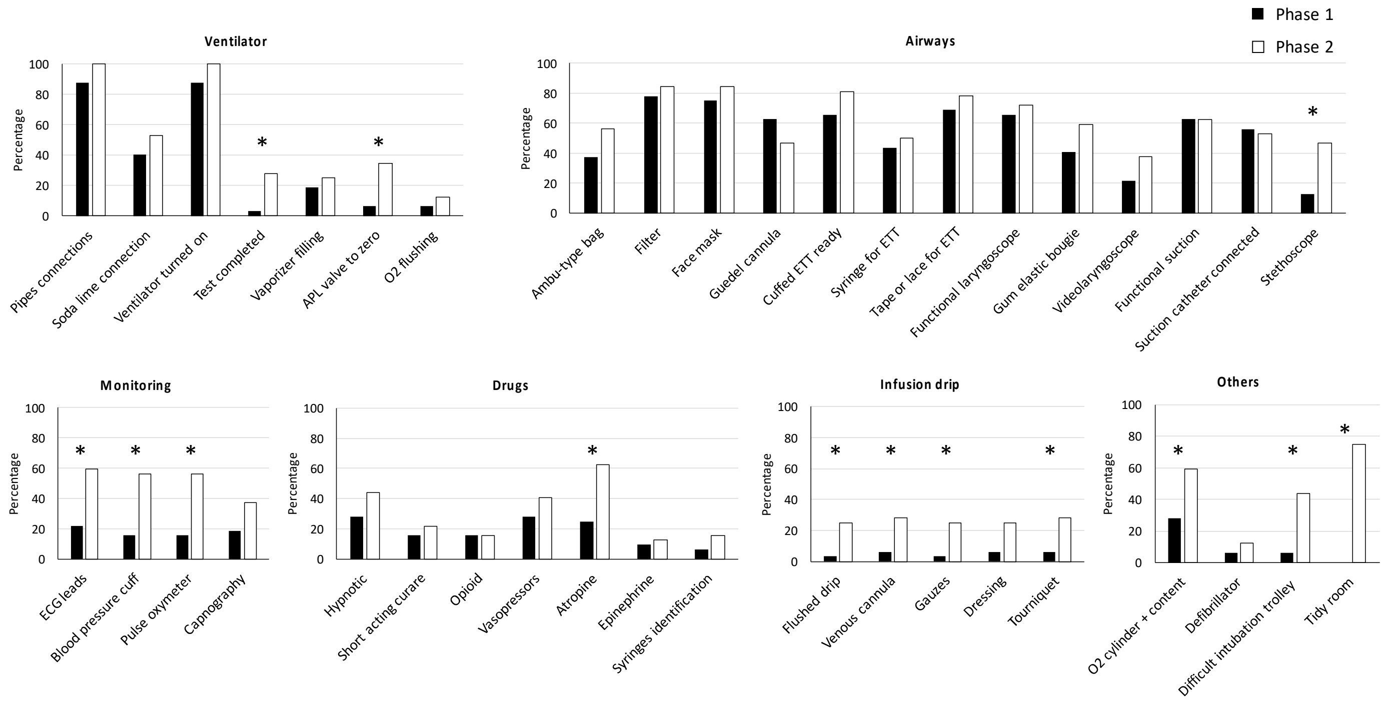

Figure S2. Percentages of residents (n = 32) who validated each required item, without time limit, in phase 1 and phase 2. *, $p < 0.05$ between the 2 phases.

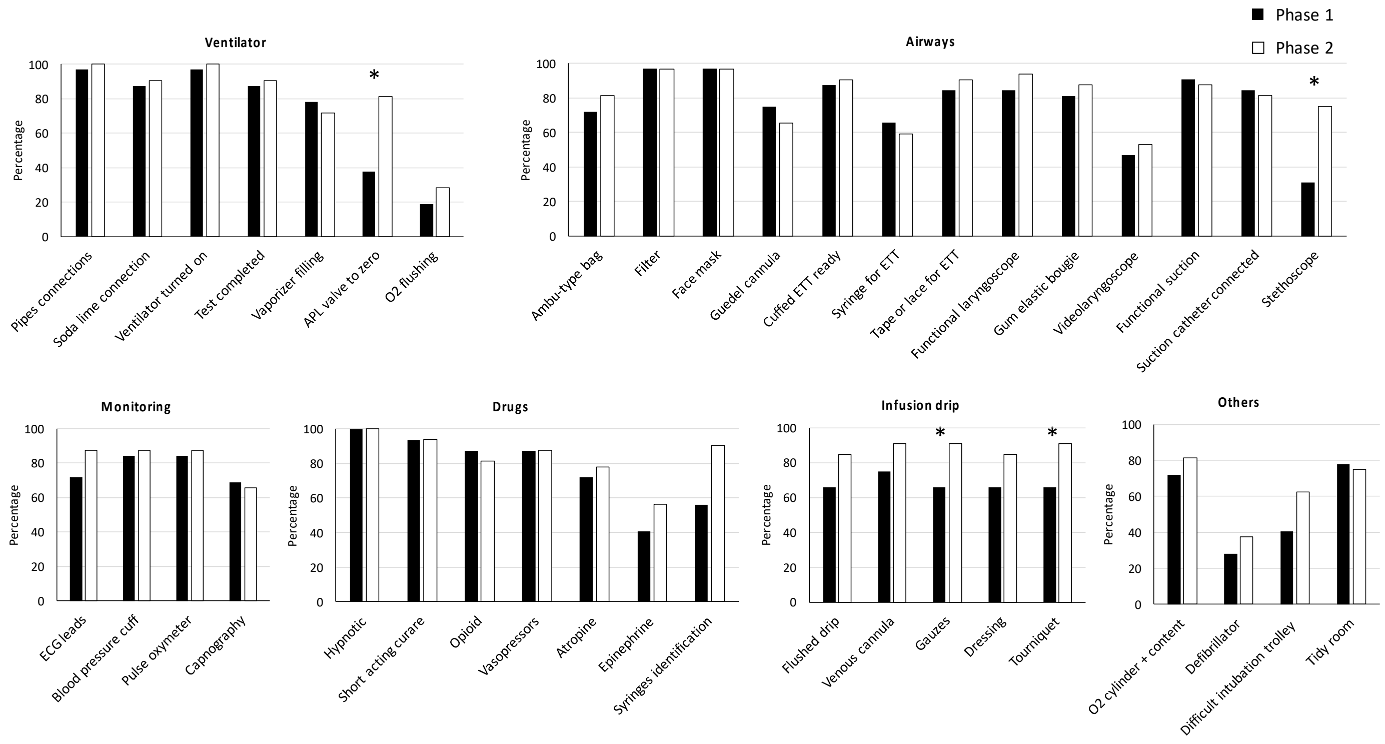

Supplement: Supplementary file 1 [file healthcare-09-01646-s001.zip › healthcare-1471530-supplementary.pdf]
